# Geographic variation in growth and wood traits of *Neolamarckia cadamba* in China

**DOI:** 10.48130/FR-2022-0012

**Published:** 2022-09-30

**Authors:** Qingmin Que, Kunxi Ouyang, Chunmei Li, Buye Li, Huiyun Song, Pei Li, Ruiqi Pian, Huaqing Li, Xiaoyang Chen, Changcao Peng

**Affiliations:** 1 College of Forestry and Landscape Architecture, Guangdong Key Laboratory for Innovative Development and Utilization of Forest Plant Germplasm, South China Agricultural University, Wushan Road 483, Tianhe District, Guangzhou 510642, P.R. China; 2 China Forestry Group Leizhou Forestry Bureau Co., Ltd., Zhanjiang 524348, Guangdong, P.R. China; 3 State Key Laboratory for Conservation and Utilization of Subtropical Agro-bioresources, South China Agricultural University, Wushan Road 483, Tianhe District, Guangzhou 510642, P.R. China

**Keywords:** growth trait, wood property, superior provenance, genetic correlation

## Abstract

*Neolamarckia cadamba* is an indigenous, timber-producing tree species in Southern China that plays an important role in the sustainable development of the local forestry industry. However, the geographic genetic variation across its natural distribution area in Southern China has yet to be characterized for best utilization. Here, we report the geographic genetic variation in growth and wood properties of *N. cadamba* from 10 provenances that represent the entire natural distribution of* N. cadamba* in Southern China. There was significant geographic variation in diameter breast height (DBH), height (H), volume (V), vessel length (VL), vessel diameter (VD), VL/VD, and wood basic density (WBD). The variation in tree volume across provenances was greater than that of other growth traits, indicating that volume has a greater potential for selection in provenance trials. The provenance heritabilities of growth traits and wood properties ranged from 0.59 to 0.67 and from 0.40 to 0.45, respectively. Trend surface analysis revealed that patterns of geographic variation associated with growth traits were weakly negatively correlated with those of wood properties. The pattern of geographic variation in growth traits showed a gradual increase from the periphery to the central region, whereas wood properties showed the opposite pattern, and latitude had the greatest effect on both. Wood property measurements suggested that the YNMS provenance produced superior timber wood, whereas the GXLZ, GXFCG, and GXNN provenances produced the best pulpwood. These provenances could potentially provide more valuable breeding materials for the genetic improvement of *N. cadamba*.

## INTRODUCTION

Forest trees are important because they provide not only timber but also raw materials for various purposes, including pulp, fuel, bioenergy feedstock, plywood, charcoal, medicines, and food. In addition to their usefulness, forest trees also contribute to terrestrial ecosystems. For example, they deliver important ecological services such as water conservation, climate regulation, biodiversity protection, and carbon recycling, and they have helped to shape the diversity of human culture^[1]^. As the human population grows exponentially, the demand for wood is increasing sharply, leading to a worldwide acceleration in deforestation. Estimates suggest that about 2 billion hectares of forests must be restored worldwide to meet these needs^[2]^, and forest ecosystems are in urgent need of protection for sustainable development. Many countries have implemented natural forest protection projects, and these are very important for protecting biodiversity and improving the ecology of many regions. Such projects can have a remarkable effect on improving our environment and alleviating possible ecological disasters; for example, the implementation of strict forest protection policy in China led to an increase in vegetation that accounted for more than 25% of the total global increase from 2000 to 2017^[3]^. However, such projects will inevitably intensify the conflict between timber demand and ecological/environmental considerations. Such a challenging problem can be addressed, to some degree, by conventional tree breeding, which can maximize the natural production potential of forest trees through genetic selection. However, the growth traits and wood properties of forest trees are polygenetic or complex traits regulated by multiple genes, which may also interact with the environment^[4]^. Climatic factors such as precipitation, temperature and light levels all play important roles in the growth of tree species. Larsen et al. found that precipitation was significantly positively correlated with the annual ring-width growth of white spruce (*Picea glauca*) and jack pine (*Pinus banksiana*)^[5]^. Nagamitsu et al. found that provenances of Japanese larch (*Larix kaempferi*) from eastern Japan, where seasonal variation in temperature is lowest and that of precipitation is highest, showed the highest performance in stem and branch growth^[6]^. To improve the yield and quality of forest products and optimize the economic and ecological benefits of forest trees, we can use strategies for the selection of germplasm resources, such as provenance tests, which enable gene pyramiding and selection of the best provenances harboring genes that positively regulate trait(s) of interest. This also allows us to take advantage of geographic genetic variation^[7]^ and match the most appropriate trees to specific sites.

*Neolamarckia cadamba* (Roxb.) Bosser (Rubiaceae)^[8]^ is an evergreen broadleaved tree species found from South Asia to Australia. It has been introduced to Surinam, South Africa, Costa Rica, and other tropical and subtropical areas^[9]^ because of its tremendous economic and ecological value^[10]^. *N. cadamba* is used not only for furniture-making but also for pulp and paper production^[11]^. Its leaves, bark, flowers, and fruit are widely used in traditional and modern medicine^[12]^. The species has also attracted significant attention for other reasons. For example, its pollen is used for apiculture^[13]^, its leaves for silage^[14]^, and the whole tree for its value in the landscape^[15]^. To date, growth traits and wood properties have been the focus of the *N. cadamba* breeding program^[16−19]^. At the World Forestry Congress in 1972, *N. cadamba* was described as 'a miraculous tree'. Under normal conditions, it can attain a height of 17.67 m and a trunk diameter of 25.3 cm at breast height within 9 years^[20]^. Therefore, it has potential, in suitable regions, to meet the increasing demand for wood products.

Progeny tests of *N. cadamba* from Southern China have been conducted in Guangdong. Preliminary results from the field trials indicate that there are significant differences between provenances and families within provenances with respect to height and diameter at breast height, and these characteristics exhibited moderate heritability (0.53–0.79)^[21,22]^. However, to date, the geographic variation among genetic breeding resources remains unexplored, and corresponding wood properties have not yet been reported. In the present study, we quantified differences in the growth and wood quality of different provenances and characterized associated patterns of geographic variation. In addition, we identified provenances that exhibited rapid growth and desirable wood properties, and we collected materials to preserve as germplasm resources and for use in future breeding activities.

## RESULTS

### Phenotypic variation in growth traits

**Figure 1:**
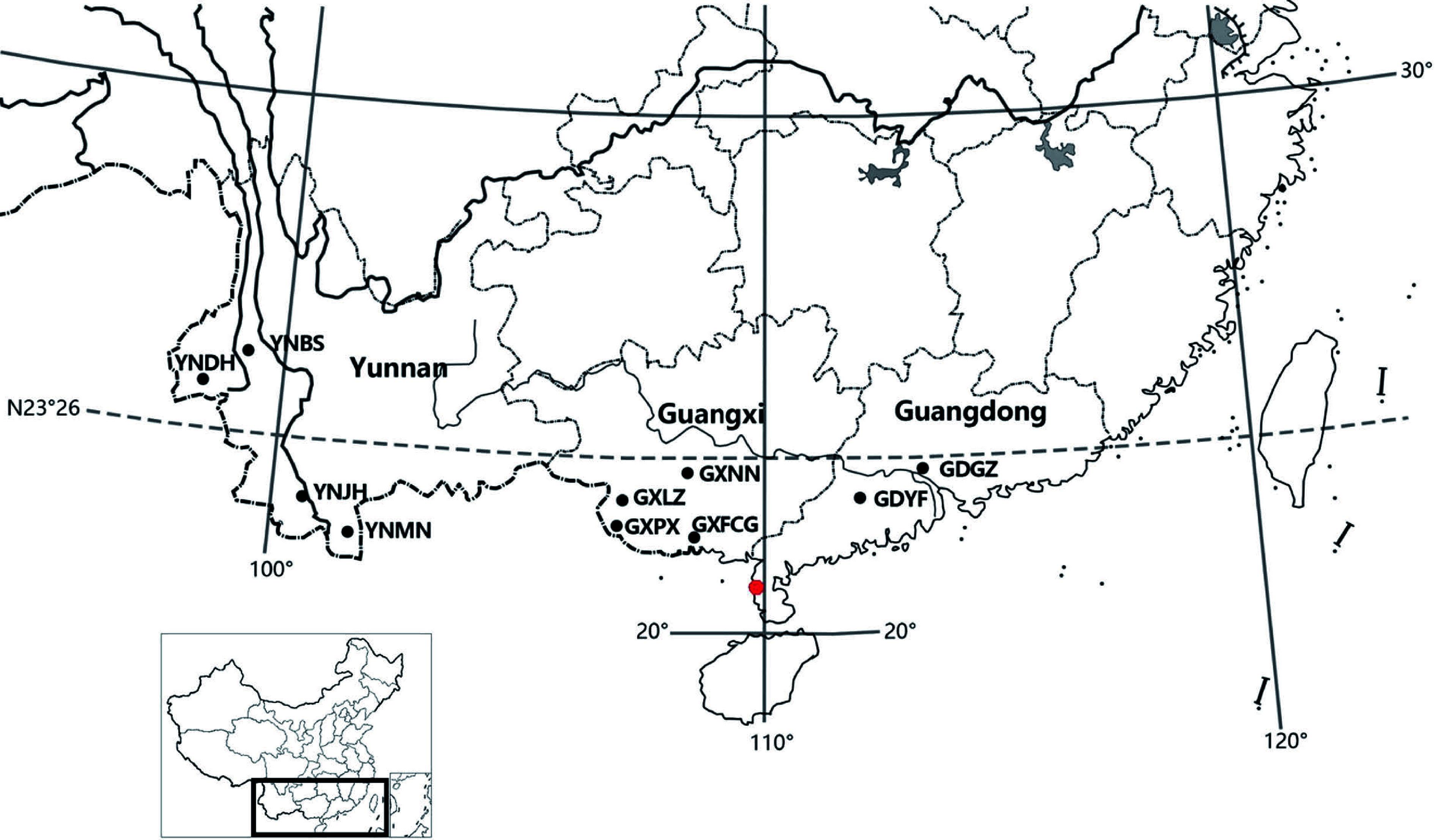
Geographic origins of the 10 *N. cadamba* provenances (black dots) and location of the test site (red dot).

Ten geographic provenances were used in this study, covering the entire natural distribution of *N. cadamba* in China (Fig. 1). The variation in all growth traits is shown in Table 1. For all traits, the degree of variation between provenances was significant (multiple comparison analysis at the *P *< 0.05 level). The provenances with the highest mean diameter at breast height (DBH), height (H), and tree volume (V) were GXFCG (13.31 cm), GXNN (13.09 m), and GXNN (0.1022 m^3^), respectively. Those with the lowest mean values of these traits were GDGZ (11.41 cm), GDGZ (10.91 m), and YNBS (0.0695 m^3^). The largest individual values of DBH, H, and V were all recorded in provenance GXFCG (20.90 cm, 19.5 m, and 0.3143 m^3^, respectively), and the smallest individual values of these traits were recorded in YNDH (3.9 cm), GXLZ (1.00 m), and YNDH (0.0022 m^3^). The ranges and coefficients of variation (CVs) are also presented in Table 1. The provenances with the greatest DBH, H, and V ranges were GXLZ (4.10–20.80 cm), GXLZ (1.00–19.00 m), and GXFCG (0.0127–0.3134 m^3^), respectively. Of all growth traits, V exhibited the highest ratio of the maximum to minimum value, reaching 24.68 fold. The YNJH provenance had the smallest range of DBH (6.50–18.20 cm), H (7.00–18.00 m), and V (0.0109–0.2128 m^3^), and the YNJH provenance had the lowest CV for all growth traits (21.94%, 23.64%, and 58.79% for DBH, H, and V, respectively), indicating low variation in growth traits for this provenance. The provenances with the highest CVs for DBH, H, and V were YNDH (31.54%), GXLZ (32.22%), and YNMN (81.63%), respectively.

**Table 1:** Comparison of growth traits among provenances of *N*. *cadamba.*

Trait	Provenance	Mean	Minimum	Maximum	CV (%)
DBH (cm)	GXLZ	12.92 ± 0.52^ab^	4.10	20.80	28.88
	GXFCG	13.31 ± 0.45^a^	7.00	20.90	24.74
	GXNN	13.26 ± 0.52^a^	4.40	20.10	28.84
	GDGZ	11.41 ± 0.49^b^	5.60	19.30	28.07
	GDYF	12.03 ± 0.56^ab^	4.80	20.10	30.12
	YNBS	11.66 ± 0.50^ab^	4.20	17.80	27.86
	YNDH	12.20 ± 0.54^ab^	3.90	19.80	31.54
	YNJH	13.22 ± 0.44^a^	6.50	18.20	21.94
	YNMS	13.01 ± 0.47^ab^	5.70	20.80	26.00
	YNMN	12.14 ± 0.55^ab^	5.20	19.70	29.86
H (m)	GXLZ	12.01 ± 0.54^abcd^	1.00	19.00	32.22
	GXFCG	12.98 ± 0.44^ab^	7.00	19.50	24.79
	GXNN	13.09 ± 0.46^a^	6.00	19.00	25.64
	GDGZ	10.91 ± 0.51^d^	4.00	17.30	30.44
	GDYF	11.28 ± 0.52^cd^	5.00	17.00	29.91
	YNBS	11.29 ± 0.56^cd^	5.00	18.00	32.74
	YNDH	11.91 ± 0.53^abcd^	4.00	19.00	31.75
	YNJH	12.78 ± 0.46^abc^	7.00	18.00	23.64
	YNMS	12.12 ± 0.48^abcd^	5.00	18.00	28.60
	YNMN	11.43 ± 0.53^bcd^	5.00	18.00	30.81
V (m^3^)	GXLZ	0.0907 ± 0.0096^ab^	0.0025	0.3033	75.40
	GXFCG	0.0991 ± 0.0091^a^	0.0127	0.3143	67.23
	GXNN	0.1022 ± 0.0095^a^	0.0043	0.2833	68.04
	GDGZ	0.0647 ± 0.0078^b^	0.0058	0.2378	78.95
	GDYF	0.0745 ± 0.0085^ab^	0.0043	0.2236	73.91
	YNBS	0.0695 ± 0.0080^b^	0.0040	0.2104	75.09
	YNDH	0.0833 ± 0.0094^ab^	0.0022	0.2504	80.04
	YNJH	0.0930 ± 0.0083^ab^	0.0109	0.2128	58.79
	YNMS	0.0890 ± 0.0079^ab^	0.0060	0.2249	64.20
	YNMN	0.0784 ± 0.0096^ab^	0.0060	0.2291	81.63
H, height; DBH, diameter at breast height; V, volume; CV, coefficient of variation. In column three, values within a trait that share lowercase superscripts are not significantly different according to Duncan's multiple range test (*p* = 0.05).					

The mean coefficients of variation for DBH, H, and V were 27.79%, 29.05%, and 72.33%, respectively, indicating substantial potential for genetic improvement of growth traits among provenances. Out of all traits, DBH had the smallest CV, and V had the largest, far greater than those of the other traits, indicating a high potential for genetic improvement in *N. cadamba*.

### Phenotypic variation in wood properties

Table 2 shows descriptive statistics for the wood properties of all provenances. Each trait exhibited a different degree of variation, and all traits except Cr differed significantly among the provenances. The GXLZ provenance had the largest mean values of FL, FD, FL/FD, VL, and VD, whereas the GDYF provenance had the smallest mean values of FL, FD, FL/FD, and VD. The largest and smallest mean values of WBD were for YNBS (0.3600 g/cm^3^) and YNDH (0.3253 g/cm^3^), respectively. The YNBS provenance had the largest range of FL and FD, and the corresponding CVs were also the largest. The largest and smallest ranges of other traits were from different provenances.

**Table 2:** Comparison of wood properties among provenances of *N. cadamba.*

Trait	Provenance	Mean	Minimum	Maximum	CV (%)
FL (μm)	GXLZ	1,525.52 ± 21.43^a^	1,442.72	1,635.32	3.97
	GXFCG	1,459.09 ± 45.27^a^	1,191.38	1,630.81	9.81
	GXNN	1,432.45 ± 23.75^ab^	1,334.34	1,555.94	5.50
	GDGZ	1,411.77 ± 38.03^ab^	1,127.74	1547.69	9.33
	GDYF	1,303.55 ± 70.03^b^	1,152.66	1,428.27	10.74
	YNBS	1,450.56 ± 35.81^a^	1,072.86	1,692.28	11.84
	YNDH	1,415.46 ± 24.41^ab^	1180.88	1,619.33	8.27
	YNJH	1,398.71 ± 21.27^ab^	1,100.28	1,661.14	9.86
	YNMS	1,403.86 ± 34.67^ab^	1,259.07	1,554.06	6.98
	YNMN	1,428.68 ± 31.09^ab^	1,184.37	1,618.10	8.70
FD (μm)	GXLZ	32.99 ± 0.40^a^	30.86	34.26	3.47
	GXFCG	32.11 ± 0.44^ab^	29.11	33.39	4.29
	GXNN	32.65 ± 0.28^a^	31.51	34.56	2.80
	GDGZ	32.60 ± 0.38^a^	29.61	34.24	4.08
	GDYF	31.35 ± 0.35^b^	30.36	32.02	2.25
	YNBS	32.31 ± 0.32^ab^	28.45	35.85	4.73
	YNDH	32.57 ± 0.21^a^	30.54	34.27	3.03
	YNJH	32.38 ± 0.18^ab^	28.66	34.20	3.67
	YNMS	31.96 ± 0.35^ab^	30.24	32.97	3.14
	YNMN	32.30 ± 0.25^ab^	30.30	33.84	3.11
FL/FD	GXLZ	46.27 ± 0.72^a^	44.18	50.27	4.40
	GXFCG	45.37 ± 0.98^a^	40.93	48.85	6.81
	GXNN	43.87 ± 0.57^ab^	41.84	47.78	4.30
	GDGZ	43.23 ± 0.74^ab^	36.96	45.73	5.94
	GDYF	41.53 ± 1.89^b^	37.97	44.99	9.10
	YNBS	44.77 ± 0.77^a^	37.71	49.41	8.24
	YNDH	43.42 ± 0.59^ab^	37.50	50.06	6.55
	YNJH	43.15 ± 0.54^ab^	36.98	50.55	8.15
	YNMS	43.90 ± 0.78^ab^	40.96	47.38	5.00
	YNMN	44.22 ± 0.86^ab^	37.16	50.98	7.82
VL (μm)	GXLZ	696.34 ± 33.29^a^	572.87	873.28	13.52
	GXFCG	656.88 ± 26.47^ab^	522.80	802.90	12.74
	GXNN	601.42 ± 20.41^b^	521.48	732.25	11.26
	GDGZ	643.94 ± 19.49^ab^	551.55	748.09	10.48
	GDYF	676.82 ± 60.44^ab^	549.34	823.63	17.86
	YNBS	691.06 ± 20.35^a^	547.53	875.98	14.12
	YNDH	651.15 ± 23.54^ab^	491.42	866.51	17.34
	YNJH	659.95 ± 12.82^ab^	532.67	833.18	12.59
	YNMS	654.00 ± 26.61^ab^	550.01	787.49	11.51
	YNMN	639.87 ± 16.40^ab^	551.43	822.21	10.25
VD (μm)	GXLZ	176.31 ± 5.97^a^	151.27	195.29	9.58
	GXFCG	164.43 ± 9.94^ab^	108.29	207.51	19.11
	GXNN	165.83 ± 6.23^ab^	140.70	203.94	12.46
	GDGZ	168.49 ± 10.19^ab^	120.39	243.96	20.95
	GDYF	144.87 ± 12.75^b^	118.55	177.27	17.60
	YNBS	158.96 ± 5.95^ab^	107.95	226.68	17.97
	YNDH	162.25 ± 6.13^ab^	111.55	231.30	18.11
	YNJH	160.13 ± 3.27^ab^	99.36	193.57	13.25
	YNMS	157.31 ± 7.60^ab^	120.70	176.66	13.67
	YNMN	165.72 ± 5.31^ab^	129.60	213.93	12.81
VL/VD	GXLZ	3.95 ± 0.12^bc^	3.57	4.60	8.73
	GXFCG	4.06 ± 0.15^bc^	3.56	4.83	11.75
	GXNN	3.65 ± 0.12^c^	3.06	4.50	10.81
	GDGZ	3.93 ± 0.19^bc^	2.86	4.90	16.33
	GDYF	4.68 ± 0.21^a^	4.16	5.17	8.90
	YNBS	4.41 ± 0.11^ab^	3.52	5.64	12.43
	YNDH	4.08 ± 0.15^bc^	3.28	5.68	17.72
	YNJH	4.16 ± 0.09^abc^	3.10	5.63	13.27
	YNMS	4.22 ± 0.24^ab^	3.12	5.19	15.88
	YNMN	3.89 ± 0.10^bc^	3.33	4.77	10.07
WBD (g/cm^3^)	GXLZ	0.3275 ± 0.0101^bc^	0.2886	0.3782	8.74
	GXFCG	0.3389 ± 0.0099^abc^	0.2878	0.3896	9.20
	GXNN	0.3274 ± 0.0081^bc^	0.2980	0.3820	8.21
	GDGZ	0.3457 ± 0.0067^abc^	0.3188	0.3900	6.69
	GDYF	0.3435 ± 0.0160^abc^	0.3146	0.3892	9.31
	YNBS	0.3600 ± 0.0046^a^	0.3302	0.4128	6.15
	YNDH	0.3253 ± 0.0048^c^	0.2656	0.3660	7.13
	YNJH	0.3381 ± 0.0042^abc^	0.2960	0.3958	8.14
	YNMS	0.3533 ± 0.0085^ab^	0.3252	0.3872	6.83
	YNMN	0.3298 ± 0.0092^bc^	0.2606	0.4000	11.13
Cr (%)	GXLZ	50.24 ± 0.89^a^	47.9	53.96	5.04
	GXFCG	50.15 ± 0.45^a^	47.44	51.81	2.87
	GXNN	52.17 ± 0.70^a^	47.84	56.36	4.43
	GDGZ	50.62 ± 0.85^a^	45.17	55.38	5.80
	GDYF	51.19 ± 0.62^a^	49.87	52.77	2.43
	YNBS	51.90 ± 0.67^a^	45.91	55.79	6.22
	YNDH	51.87 ± 0.47^a^	47.93	58.23	4.33
	YNJH	52.10 ± 0.47^a^	40.95	61.11	5.87
	YNMS	52.62 ± 0.84^a^	50.05	57.39	4.50
	YNMN	50.63 ± 0.76^a^	45.73	56.01	6.00
FL, fiber length; FD, fiber diameter; FL/FD, the ratio of FL to FD; VL, vessel length; VD, vessel diameter; VL/VD, the ratio of VL to VD; WBD, wood basic density; Cr, Degree of crystallinity; CV, coefficient of variation. In column three, values within a trait that share lowercase superscripts are not significantly different according to Duncan’s multiple range test (*p* = 0.05).					

The mean CVs for FL, FD, FL/FD, VL, VD, VL/VD, WBD, and Cr were 8.50%, 3.46%, 6.63%, 13.17%, 15.55%, 12.59%, 8.15%, and 4.75%, respectively. Among these traits, the largest and smallest CVs were for VD and FD, respectively. The mean CVs for vessel traits were all greater than 10%, indicating that the potential for genetic improvement among provenances was greater for these traits than for the other wood properties investigated.

### Differences in growth and wood property traits among provenances

The variance components, provenance heritabilities (*h*^*2*^), and genetic variation coefficients (*CV*_*G*_) among the provenances were estimated (Table 3). The provenance variance components (*V*_*P*_) for DBH, H, and V accounted for 4.97%, 3.87%, and 4.33% of the corresponding total variance, respectively. The provenance by block interaction variance components (*V*_*PB*_) for DBH, H, and V accounted for 0.84%, 4.37%, and 1.47% of the corresponding total variance, respectively. The *V*_*PB*_ percentages for DBH and V were lower than the corresponding *V*_*P*_ percentages, whereas the opposite was true for H. This suggests that interactions between provenance and block were lower for DBH and V than for H. The variance components of provenance were always lower than the variance components associated with random error (*V*_*e*_), suggesting that random environmental effects could be a major cause of variation in the studied traits, whereas the genetic effects were more limited. The provenance heritabilities of DBH, H, and V were 0.67, 0.59, and 0.64, respectively. This indicates that growth traits of *N. cadamba* are under moderate to high genetic control. By contrast, the provenance heritabilities for wood properties were in the range of 0.02–0.45, suggesting that these properties were under low to moderate genetic control. The differences in variance for FL, FD, FL/FD, VL, VD, and Cr among the provenances were not significant (the standard error was much larger than the corresponding estimated value).

**Table 3:** Variance components, provenance heritabilities, and genetic variation coefficients among the provenances.

Trait	*V*_*P*_ (SE)	*V*_*PB*_ (SE)	*V*_*e*_ (SE)	*h* ^*2*^	*CV*_*G*_ (%)
DBH	0.47 (0.22)	0.08 (0.07)	8.97 (0.32)	0.67	5.46
H	0.23 (0.14)	0.26 (0.10)	5.46 (0.19)	0.59	4.00
V	9.98e−05 (5.91e−05)	3.39e−05 (2.64e−05)	2.17e−03 (7.85e−05)	0.64	11.83
FL	69.50 (533.00)	NE	17,595.30 (2037.00)	0.02	0.59
FD	0.00 (0.03)	NE	1.41 (0.16)	NE	NE
FL/FD	0.31 (0.46)	NE	10.03 (1.16)	0.16	1.26
VL	94.03 (277.50)	NE	7,771.29 (900.80)	0.07	1.48
VD	0.00 (17.78)	NE	644.00 (74.50)	NE	NE
VL/VD	3.52e−02 (2.81e−02)	NE	0.31 (0.03)	0.40	4.57
WBD	1.01e−04 (7.48e−05)	NE	7.35e−04 (8.56e−05)	0.45	2.96
Cr	0.16 (0.31)	NE	7.61 (0.88)	0.11	0.78
H, height; DBH, diameter at breast height; V, volume; FL, fiber length; FD, fiber diameter; FL/FD, the ratio of FL to FD; VL, vessel length; VD, vessel diameter; VL/VD, the ratio of VL to VD; WBD, wood basic density; Cr, degree of crystallinity; *V*_*P*_, provenance variance; *V*_*PB*_, provenance by block interaction variance; *V*_*e*_, random error variance;* h*^*2*^, provenance heritability; *CV*_*G*_ genetic variation coefficient; SE, standard error. NE, not estimated and assumed to be zero.					

For traits with a significant *V*_*P*_, the genetic variation coefficient was in the range 0.59%–11.83%. Only the* CV*_*G*_ of V was greater than 10%, indicating that the provenance selection potential of V is greater than that of the other traits.

### Analysis of phenotypic and genetic correlations

The genetic and phenotypic correlations between all studied traits are presented in Table 4 (the genetic correlations for FD and VD were not estimated because the corresponding provenance variances were zero). The genetic and phenotypic correlations between growth traits (DBH, H, and V) were large, positive, and significant (0.97–0.99 for genetic correlations, 0.87–0.98 for phenotypic correlations), and the genetic correlations were always larger than the corresponding phenotypic correlations. Low to moderate genetic correlations between wood properties were observed, except between FL and FL/FD. In the analysis of phenotypic correlations between wood properties, the correlations between Cr and other wood properties were always low (absolute values of the correlation coefficients were in the range 0.02–0.18). The correlations between WBD and other wood properties were also low (absolute values 0.02–0.28), except for those with FD (−0.52) and VL (0.99). There were significant moderate to high positive correlations between fiber traits and vessel traits (0.41–0.99), except for the correlation between VL/VD and FL (0.28) and the correlation between VL/VD and FL/FD (0.20). The genetic correlations between growth traits and wood properties were always low (absolute values 0.02–0.22) and negative (except for those between H and FL and between H and FL/FD), and the corresponding standard errors were always larger than the estimated correlation coefficients (except for those of correlation coefficients between WBD and growth traits). The phenotypic correlations between growth traits and wood properties were low to moderate (absolute values 0.07–0.41). The phenotypic correlations between growth traits and fiber/vessel traits were significant and positive (except for those between growth traits and VL/VD). The phenotypic correlations between growth traits and WBD or Cr were weak and negative.

**Table 4:** Genetic (*r*_*g*_) and phenotypic (*r*_*P*_) correlations between all studied traits.

	DBH (SE)	H (SE)	V (SE)	FL (SE)	FD (SE)	FL/FD (SE)	VL (SE)	VD (SE)	VL/VD (SE)	WBD (SE)	Cr (SE)
DBH		0.97 (0.01)	0.99 (0.01)	−0.07 (0.27)	NE	−0.21 (0.69)	−0.22 (0.24)	NE	−0.05 (0.15)	−0.17 (0.16)	−0.02 (0.42)
H	0.87 (0.01)		0.97 (0.02)	0.10 (0.37)	NE	0.01 (0.19)	−0.18 (0.26)	NE	−0.03 (0.12)	−0.16 (0.12)	−0.02 (0.34)
V	0.98 (0.01)	0.91 (0.05)		−0.09 (0.19)	NE	−0.15 (0.39)	−0.15 (0.30)	NE	−0.02 (0.13)	−0.18 (0.15)	−0.03 (0.41)
FL	0.34 (0.07)	0.39 (0.07)	0.36 (0.07)		NE	0.99 (0.01)	0.58 (0.82)	NE	0.17 (0.22)	0.01 (0.45)	−0.14 (0.17)	
FD	0.32 (0.07)	0.32 (0.07)	0.30 (0.07)	0.71 (0.04)		NE	NE	NE	NE	NE	NE
FL/FD	0.28 (0.08)	0.34 (0.07)	0.30 (0.07)	0.99 (0.01)	0.41 (0.07)		−0.02 (0.58)	NE	0.17 (0.83)	0.46 (0.79)	−0.41 (0.68)
VL	0.17 (0.08)	0.18 (0.08)	0.16 (0.08)	0.60 (0.05)	0.70 (0.10)	0.51 (0.06)		NE	0.66 (0.01)	0.44 (0.56)	0.09 (0.69)	
VD	0.41 (0.07)	0.39 (0.07)	0.38 (0.07)	0.70 (0.04)	0.99 (0.07)	0.56 (0.06)	0.47 (0.23)		NE	NE	NE
VL/VD	−0.35 (0.07)	−0.31 (0.09)	−0.31 (0.08)	−0.28 (0.08)	−0.53 (0.10)	−0.20 (0.08)	0.57 (0.16)	−0.66 (0.05)		0.02 (0.19)	0.87 (0.82)
WBD	−0.12 (0.08)	−0.11 (0.08)	−0.10 (0.08)	−0.14 (0.08)	−0.52 (0.10)	−0.02 (0.08)	−0.99 (0.86)	−0.28 (0.07)	0.20 (0.09)		0.29 (0.81)
Cr	−0.12 (0.08)	−0.08 (0.08)	−0.07 (0.08)	−0.02 (0.12)	−0.06 (0.08)	0.02 (0.12)	0.07 (0.08)	−0.15 (0.12)	0.18 (0.08)	0.14 (0.08)	
Above the diagonal are genetic correlations, and below the diagonal are phenotypic correlations. H, height; DBH, diameter at breast height; V, volume; FL, fiber length; FD, fiber diameter; FL/FD, the ratio of FL to FD; VL, vessel length; VD, vessel diameter; VL/VD, the ratio of VL to VD; WBD, wood basic density; Cr, degree of crystallinity; SE, Standard error. NE, not estimated and assumed to be zero.											

### Geographic patterns

To better understand possible trends associated with geographic patterns, we performed a binary quadratic trend surface analysis (Table 5). The regression equations for DBH, H, V, VL/VD, and WBD were significant, whereas those for FL, FD, FL/FD, VL, VD, and Cr were not, and subsequent discussions therefore focus on the former traits.

**Table 5:** Regression equations obtained by binary quadratic trend surface analysis.

Trait	Regression equation of trend surface analysis	Fitting coefficient	*p-*value
DBH	*Z* = −393.1 + 14.85*x* + 4.574*y* − 0.3657*x*^2^ − 0.02434*y*^2^ + 0.01936*xy*	0.0195	7.34e−07
H	*Z* = −169.6 + 7.012*x* + 1.971*y*−0.2409*x*^2^− 0.01412*y*^2^ + 0.04008*xy*	0.0153	1.68e−05
V	*Z* = −5.54 + 0.2083*x* + 0.06306*y* − 0.004804*x*^2^ − 0.0003198*y*^2^ + 0.0001185*xy*	0.0171	4.73e−06
FL	*Z* = −16750 + 145.6*x* + 309.1*y* − 7.195*x*^2^ − 1.695*y*^2^ + 2.044*xy*	0.0409	0.2709
FD	*Z* = 76.805095 − 2.054393*x* − 0.447867*y* − 0.099681*x*^2^ − 0.005207*y*^2^ + 0.06647*xy*	0.0226	0.6524
FL/FD	*Z* = −587.30009 + 7.92364*x* + 10.20365*y* − 0.11231*x*^2^ − 0.04611*y*^2^ − 0.02193*xy*	0.0578	0.1059
VL	*Z* = −691.3203 − 145.7474*x* + 58.2785*y* + 11.404*x*^2^ + 0.1355*y*^2^ − 3.7184*xy*	0.0372	0.3282
VD	*Z* = −531.15 + 12.6525*x* + 9.5999*y* − 2.3297*x*^2^ − 0.1494*y*^2^ + 0.9469*xy*	0.0230	0.6162
VL/VD	*Z* = 25.438914 − 1.402947*x* − 0.076717*y* + 0.135262*x*^2^ + 0.005649*y*^2^ − 0.047789*xy*	0.1181	0.0018
WBD	*Z* = 4.142 − 0.3356*x* − 0.002205*y* + 0.00756*x*^2^ + 0.00002628*y*^2^ − 0.0000636*xy*	0.0272	0.0007
Cr	*Z* = 305.50611 − 11.71736*x* − 2.30488*y* + 0.06669*x*^2^ + 0.00115*y*^2^ + 0.08674*xy*	0.0309	0.4412
H, height; DBH, diameter at breast height; V, volume; FL, fiber length; FD, fiber diameter; FL/FD, ratio of FL to FD; VL, vessel length; VD, vessel diameter; VL/VD, ratio of VL to VD; WBD, wood basic density; Cr, degree of crystallinity.			

**Figure 2:**
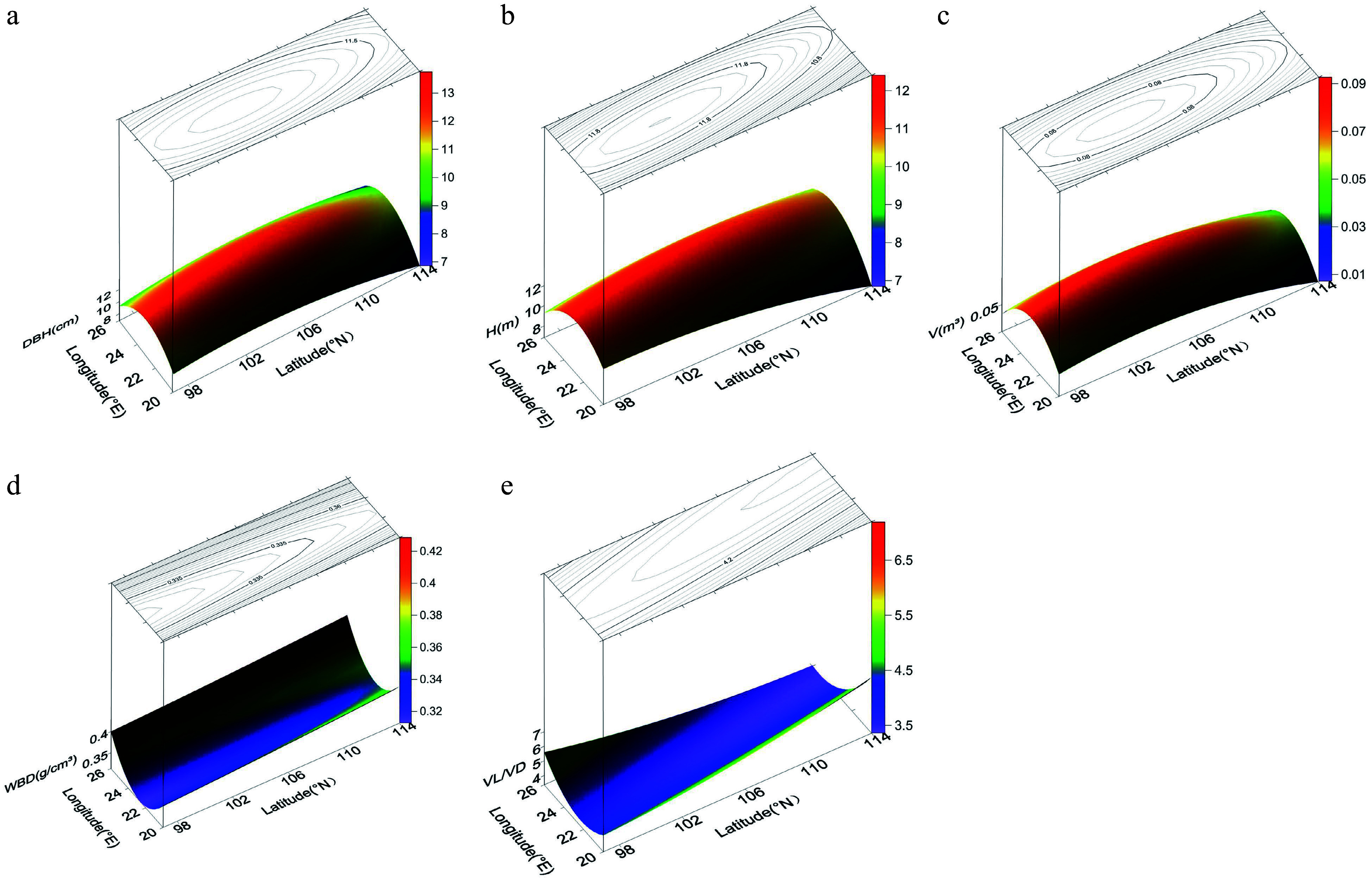
Power contour-trend surfaces for (a) diameter breast height (DBH), (b) height (H), (c) volume (V), (d) wood basic density (WBD), and (e) vessel length/vessel diameter (VL/VD) of* N. cadamba*. The surface represents geographic variation, and the lines on the surface represent contours.

The trend surface diagrams (Fig. 2) revealed that the patterns of geographic variation in growth traits were basically opposite to those in wood properties. All three growth traits (DBH, H, and V) displayed the same pattern of geographic variation: a gradual increase from the periphery to the central region. Thus, as latitude and longitude increased, the growth traits first increased then decreased. Variation was lower in the central region than at the periphery, and latitude had a greater impact on growth traits than longitude. Unlike the growth traits, WBD showed a gradual decrease from the north and south to the central region; as latitude increased, WBD first decreased then increased, and as longitude increased, WBD exhibited a slight increase. The trend for VL/VD was also different, showing a gradual decrease from the southeast and northwest to the central region. Geographic variation in VL/VD was affected by latitude more than by longitude.

### Assessment of provenance performance

**Figure 3:**
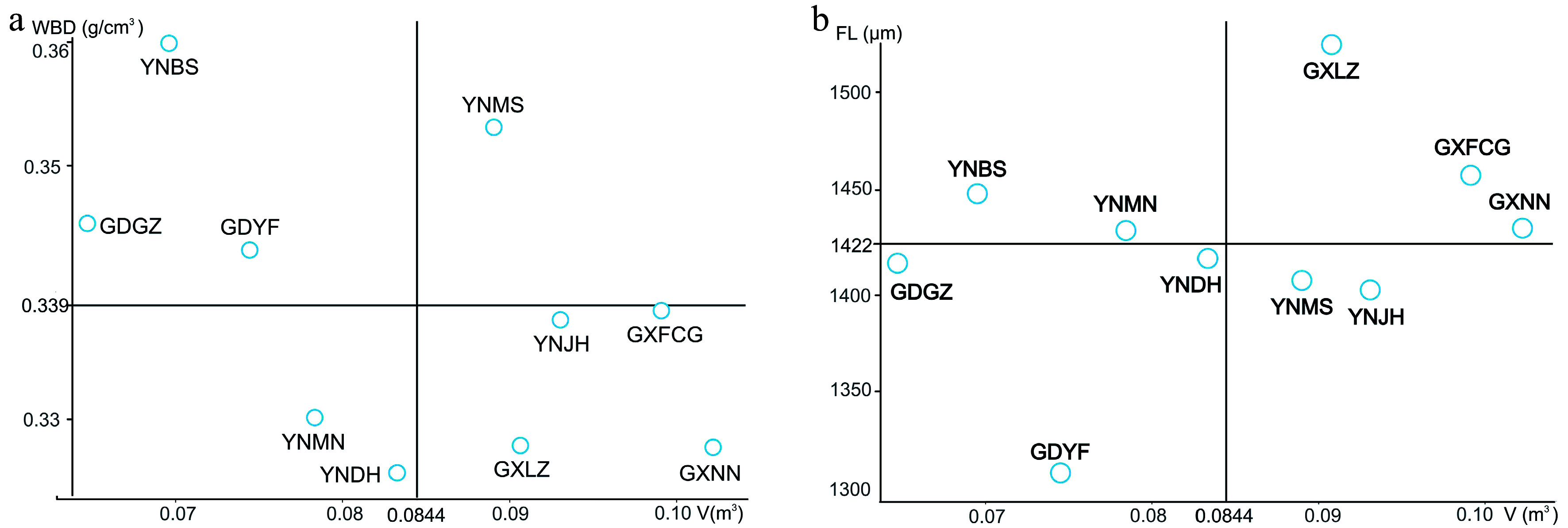
Selection of superior provenances for wood products (a) and pulpwood (b).

The main goal of forest tree breeding is to select superior provenances with rapid growth and high wood density; however, forest trees intended for different purposes should be assessed on the basis of different indicators. In the present study, we used V and WBD as indicators of wood product value and V and FL as indicators of good pulpwood sources. Taking the overall means of the relevant traits as thresholds for the selection of superior provenances for wood products, we identified five provenances for V (mean 0.0844 m^3^) and four for WBD (mean 0.3390 g/cm^3^). However, only one provenance—YNMS—had values that surpassed both thresholds (Fig. 3a). The realized gains (G) of this provenance for all studied traits are shown in Table 6. The largest realized gain was for V (12.50%), and the smallest was for VD (−3.15%). The realized gains for growth traits and VL/VD, WBD, and Cr were positive, whereas those for other fiber traits and vessel traits were negative.

**Table 6:** Realized gains in all studied traits based on selection of superior *N. cadamba* provenances using the overall mean of each trait as a threshold.

Purpose			Wood products			Pulpwood	
Trait	Overall mean		Mean value of superior provenance	G (%)		Mean value of superior provenances	G (%)
DBH (cm)	12.52 ± 0.22		13.01 ± 0.47	3.91		13.16 ± 0.12	5.11
H (m)	11.98 ± 0.24		12.12 ± 0.48	1.17		12.69 ± 0.34	5.93
V (m^3^)	0.08 ± 0.00		0.09 ± 0.01	12.50		0.10 ± 0.00	25.00
FL (μm)	1,422.97 ± 17.67		1,403.86 ± 34.67	−1.34		1,472.35 ± 27.67	3.47
FD (μm)	32.32 ± 0.14		31.96 ± 0.35	−1.11		32.58 ± 0.26	0.80
FL/FD	43.97 ± 0.41		43.90 ± 0.78	−0.16		45.17 ± 0.70	2.73
VL (μm)	657.14 ± 8.63		654.00 ± 26.61	−0.48		651.55 ± 27.53	−0.85
VD (μm)	162.43 ± 2.60		157.31 ± 7.60	−3.15		168.86 ± 3.75	3.96
VL/VD	4.10 ± 0.09		4.22 ± 0.24	2.93		3.89 ± 0.12	−5.12
WBD (g/cm^3^)	0.34 ± 0.00		0.35 ± 0.01	2.94		0.33 ± 0.00	−2.94
Cr (%)	51.35 ± 0.28		52.62 ± 0.84	2.47		50.85 ± 0.66	−0.97

For pulpwood, selection yielded five provenances with mean V values greater than the overall V mean (0.0844 m^3^) and five provenances with mean FL values greater than the overall FL mean (1,422.97 μm). Three provenances—GXLZ, GXFCG, and GXNN—had values that surpassed both thresholds (Fig. 3b). The realized gains (G) of these provenances for all studied traits are shown in Table 6. The largest realized gain was in V (25.00%), and other growth traits also had relatively high gains (5.11% and 5.93% for DBH and H, respectively), whereas the smallest gain was in VL/VD (−5.12%). The traits that are beneficial for pulpwood had moderate realized gains (3.47% and 2.73% for FL and FL/FD, respectively).

## DISCUSSION

### Genetic variation in growth traits and wood properties of *N. cadamba*

Yield per unit volume is a key indicator in forestry production, and individual tree volume is the most important indicator for predicting plantation yield potential. In the present study, all growth traits differed significantly between provenances, and the coefficient of variation for V was significantly greater (*p* < 0.001) than those for DBH and H, with the genetic variation coefficient exhibiting the same pattern. Similar results have been found in other studies that examined provenances of *N. cadamba*^[23]^ and other forest tree species such as *Betula alnoides*^[24]^, black spruce^[25]^, and Chinese Fir^[26]^. These results suggest that, in most forest tree species, the variation in tree volume between provenances is greater than that of other growth traits, thus indicating that volume has greater potential for selection in provenance trials. The provenance heritability of different growth traits was in the range 0.59–0.67, similar to the results of other provenance experiments involving *N. cadamba*^[22,23]^. This result suggests that the growth traits of *N. cadamba* are under moderate to high genetic control.

As one of the most important indicators of the physical properties of wood, wood density has a great influence on the final yield and quality of forest trees. We measured the wood properties of *N. cadamba*, including basic density, fiber properties, and vessel properties. The range of WBD was 0.26–0.41 g/cm^3^, similar to that found by Krisnawati et al.^[27]^ in Malaysia, who reported wood density (with 15% moisture content) in the range 0.29–0.56 g/cm^3^. Another study from Indonesia^[28]^ produced similar results (0.259–0.606 g/cm^3^). Fiber length and the ratio of fiber length to fiber diameter are the two most important indices for pulp quality^[29]^. In the present work, FL and FL/FD were in the ranges 1,072.86–1,692.28 μm and 36.98–50.99, respectively. Rahayu et al.^[28]^ reported similar results with respect to fiber length (800–1,700 μm). There were some differences in wood properties (except for Cr) between provenances, but only those for WBD and VL/VD were significant. Wood properties not only vary between tree species, provenances, and families but also radially within a single plant. At the same time, they tend to be correlated with the growth rate of DBH, although this occurs mainly in fast-growing tree species. The wood properties of Scots pine (*Pinus sylvestris*) differ significantly between families^[30]^, the wood density of poplars (*Populus* spp.) differs significantly between clones^[31]^, and the wood density of *Larix kaempferi* differs significantly from the pith to the bark^[32]^. In *N. cadamba*, radial growth rate can also affect wood properties^[28]^. In the present study, however, the data we used were average values for provenances, and this may be why the provenance variance of some wood traits was not significant.

### Genetic and phenotypic correlations between traits

In this study, the genetic and phenotypic correlations between growth traits were always strong and positive, as reported in other tree species^[26,31,33]^. Such results may provide a theoretical basis for the indirect selection of growth traits. In previous literature, the correlation between growth traits and wood quality in conifers has always been found to be negative^[34]^. For example, the genetic correlation between diameter and density was −0.61 for *Picea abies*^[35]^, −0.48 for *Pinus radiata*^[36]^, and −0.34 for Chinese fir^[26]^. However, in broadleaved tree species, some researchers have found the genetic correlation between growth traits and wood quality to be weak. For example, the genetic correlation between tree height and density of* Eucalyptus grandis* declined towards zero with age^[37]^, and the genetic correlation between diameter and density of *Populus tomentosa* was −0.05^[38]^. Similarly, the genetic correlations between growth traits and wood properties were weak in our study, suggesting that there is weak correlation between the genes controlling growth traits and wood quality. This result indicates that growth traits and wood traits can be selected independently when identifying superior provenances of *N. cadamba.*

### Patterns of geographic variation in *N. cadamba* provenances from southern China

The geographic variation associated with the provenance of forest trees is one of the main components of variation at different levels within a tree species. To make use of this variation, provenance experiments can be used to study the effects of geographic variation on growth traits, wood quality, stress resistance, and so forth, thus identifying high-yielding, high-quality, and stable germplasm resources for forest production. Several studies from Southeast Asia have shown that there is significant variation among provenances of *N. cadamba*^[23,39,40]^. In the present study, significant geographic variation was found in DBH, H, V, VL/VD, and WBD. However, the patterns of geographic variation associated with growth traits were the opposite of those for wood quality. This result may be related to the negative correlation between them, although this correlation was weak. Latitude had a greater influence on the traits than longitude, and the growth traits first increased and then decreased with increasing latitude. Similar results were reported in a study of Chinese fir^[41]^. This pattern is closely related to the annual average temperature and precipitation in the original distribution area of the trees. Compared with the periphery of the natural range of* N. cadamba*, the central region has a relatively high annual average temperature and a more suitable precipitation regime. Because *N. cadamba* is very sensitive to low temperatures, the frostless period and minimum temperature are also major causes of geographic variation, and the central region has a relatively longer frostless period and higher minimum temperature than the periphery. On the other hand, *Diaphania glauculalis* is an important pest that attacks *N. cadamba*, and its damage period occurs mainly during the hot and humid season^[42]^; such damage may influence growth indirectly. This may also be one of the main reasons for the pattern of geographic variation in *N. cadamba* growth traits. Thus, in addition to target traits such as growth and wood traits, resistance traits such as cold resistance and insect resistance should also be considered in the selection of superior *N. cadamba* provenances.

### Provenance selection for wood products and pulpwood

Our analysis showed that the provenances with high growth traits did not necessarily have the best wood properties. Moreover, there was a negative genetic correlation between growth traits and wood properties, although this correlation was weak. This finding highlights the need for careful consideration when selecting superior provenances, rather than simply looking at the performance of a single trait. Taking account of the different intended uses, we selected WBD and V as selection indicators for wood products and FL and V for pulpwood. YNMS was selected as an excellent provenance for the cultivation of wood products. Compared with the overall average, V had the highest realized gain (12.50%), followed by DBH (3.91%). Among the wood properties, WBD, Cr, and VL/VD also showed moderate realized gain (2.94%, 2.47%, and 2.93%, respectively), whereas the realized gains for other wood properties were negative. Provenances GXLZ, GXFCG, and GXNN were selected as being superior for pulpwood: the realized gain for V was 25.00%, and other growth traits also had relatively high gains (5.11% and 5.93% for DBH and H, respectively). Traits that are beneficial for pulpwood production all showed moderate realized gains (3.47% and 2.73% for FL and FL/FD, respectively). Similar results were found in selection of Chinese fir^[26]^; when constraining selection to DBH and WBD, growth traits and WBD increased dramatically, and hygroscopicity and tracheid diameter exhibited negative realized gains. In a breeding program for *Pinus kesiya*^[43]^, the gain in volume was found to be −9.78% when selecting only for the single trait of wood density. However, when selection was constrained to both DBH and WBD, there were simultaneous increases in strength and volume. Similar research results were also found in breeding programs for broadleaved tree species. In poplar^[44]^, when wood density or volume was selected alone, the genetic gain of another character was zero or even negative. In* Eucalyptus dunnii*, when a single trait was used as the selection criterion for pulp production^[45]^, the elite clones selected were different each time. In the long term, the best selection strategy may be to develop a multiple-trait selection index for each application. The selection of superior provenances will inevitably lead to a narrowing of genetic diversity^[6]^. Therefore, it is necessary to continuously supplement new superior provenances in the breeding population.

## CONCLUSIONS

Our results revealed that growth traits and most wood properties of *N. cadamba* differed among provenances in southern China, and the growth traits were under moderate genetic control. There were strong positive correlations between growth traits, whereas the correlations between growth traits and wood properties were weak. The patterns of geographic variation differed between growth traits and wood properties, although latitude had the greatest influence on both. The YNMS provenance was suitable for selecting superior timber-wood trees, and the GXLZ, GXFCG, and GXNN provenances showed promise for pulpwood trees. However, in the long term, a multiple-trait selection index for *N. cadamba* should be considered for different applications, including the production of medicine, juice, nectar, and silage, as well as the species' value as a landscape tree. Multi-site provenance experiments are also required to further refine our understanding of tree–site interactions and better match provenances with sites.

## MATERIALS AND METHODS

### Materials

The data were collected from a half rotation-aged progeny trial in Leizhou (21°10′06″ N, 110°21′34″ E), Guangdong province. The site has an annual mean temperature of 22 °C and annual rainfall of 1711.6 mm. The minimum and maximum temperature in this region are 15.5 and 28.4 °C, respectively. The experimental plantation was established in the spring of 2014. The experimental design in the field comprised randomized complete blocks, with 10 blocks and 5-tree plots in a 3 m × 3 m square spacing. Ten geographic provenances were planted in the trial, covering the entire natural distribution of *N. cadamba* in Southern China (Table 7). The sampled trees were chosen because they were considered phenotypically average or above average with respect to stem DBH and total height compared with neighboring trees in the population. The distance between mother trees within the population was a minimum of 100 m to reduce genetic relatedness between seed lots. Seeds were collected by climbing the trees. The seed lots from each tree were kept separate, and their location and number of samples were recorded. Dr. Mingxuan Zheng undertook the formal identification of the plant material used in our study, and the plant specimens are housed in the herbarium of South China Agricultural University (CANT32205).

**Table 7:** Geographic locations of the sampled *N. cadamba* populations and their climatic properties

Provenance	Latitude (°N)	Longitude (°E)	Altitude (m)	Annual average temperature (°C)	Minimum temperature (°C)	Maximum temperature (°C)	Frostless period (d)	Average annual precipitation (mm)
GXLZ	22.36	106.84	269	22.2	0.8	39.9	352	1,260
GXFCG	21.77	107.35	235	21.8	1.4	37.8	360	2,512
GXNN	22.85	108.4	80	21.7	−2.4	40.4	364	1,304.2
GDGZ	23.1	113.21	10	22.1	0	39.3	346	1,696.5
GDYF	22.1	112.02	346	21.5	−1	39.1	345	1,670.5
YNBS	25.08	99.16	1670	17.4	−4.2	40.4	283	1,710
YNDH	24.08	97.39	780	18.9	−2.9	35.7	299	1,544
YNJH	21.02	101.04	552.7	21	2.7	41.1	365	1,197
YNMS	24.2	98.95	913	19.6	−0.6	36.2	315	1,650
YNMN	21.4	101.3	631	21	0.5	38.4	331	1,540

### Data collection

Diameter at breast height (DBH in cm, 1.3 m above ground level) and height (H in m) of all trees were recorded. Individual tree volume (V in m^3^) was calculated using the following formula^[20]^:

1 $ V = 3.69 \times {10^{ - 5}} \times DB{H^2} \times H $

Five average trees were selected from each provenance based on DBH. From each, a 5.02-mm core was extracted at breast height using a tree growth cone. Wood basic density (WBD in g·cm^−3^) was determined using the water displacement method^[46]^ based on two measurements: volume of water displaced by immersion of the wedge (*w*_*1*_) and oven-dry weight (*w*_*2*_). Wood basic density was then calculated using the formula:

2 $ WBD = \frac{{{w_2}}}{{{w_1}}} $

Vessel length (VL), vessel diameter (VD), the ratio of VL to VD (VL/VD), fiber length (FL), fiber diameter (FD), and the ratio of FL to FD (FL/FD) were determined following Chen and Xie^[47]^. Each sample was placed in a test tube, and chromic acid-nitric acid separation solution equivalent to 10–20 times the sample volume was added; the mixture was then boiled over an alcohol lamp for 5–8 min. A small sample of the material was removed and placed on a glass slide, then pressed lightly with tweezers to determine whether it disintegrated. If not, heating was continued for another 2–3 minutes. After rinsing thoroughly with clean water, a small amount of 0.5% safranin aqueous solution was added, and the material was mashed with a glass rod. Samples were then examined under an optical microscope. Each sample was measured three times, and a total of > 30 values were obtained.

The degree of crystallinity (Cr) was measured by X-ray diffraction following Segal et al.^[48]^. The diffraction intensity of wood fiber is at its maximum value at 2*θ *= 22°, and its integrated intensity is defined as I_u_. The wave trough appears near 2*θ *= 18°, which is the scattering intensity of the diffraction in the amorphous area of the wood fiber, and its integrated intensity is defined as I_a_. Crystallinity is then calculated using the formula:

3 $ Cr = \frac{{{I_u} - {I_a}}}{{{I_u}}} \times 100{\%} $

### Statistical analysis

R version 4.0.2^[49]^ and Microsoft Excel 2013 were used to analyze variation in growth traits and wood properties, including the mean value, standard error, amplitude, and CV. To determine the differences between phenotypic variables of the provenances, Duncan’s multiple range tests were performed using the agricolae package^[50]^ in R version 4.0.2. Variance and covariance components for genetic analyses were estimated using the sommer package^[51]^ in R version 4.0.2 based on a mixed linear model:

4 $ y = X\beta+Zu+\varepsilon  $

where *y* is a vector of trait phenotypes, *β* is a vector of fixed effects (block), *u* is a vector of random effects (provenance, provenance by block interaction), and ε is the residual. *X* and *Z* are incidence matrices for fixed and random effects, respectively.

To estimate the degree of genetic control for each trait, provenance heritability (*h*^*2*^) was calculated for all traits in the provenances overall using the following formula, based on the variance component estimates from the model analyses:

5 $h^2=\frac{V_p}{V_e/n_hb+V_{PB}/b+V_p}$

where *V*_*P*_ is the provenance variance, *V*_*PB*_ is the provenance by block interaction variance, *V*_*e*_ is the random error variance, *n*_*h*_ is the adjusted number of trees, and *b* is the number of blocks.

Because some of the poorly adapted saplings died, the number of trees in each block was not constant, and the data were unbalanced. Thus it was necessary to replace the original number of trees with the actual number of trees in each plot^[52]^:

6 $ {n_h} = \left( {bp} \right)\Bigg/\sum\limits_{i = 1}^b {\sum\limits_{j = 1}^p {\left( {1/{n_{ij}}} \right)} } $

Where *p* is the number of provenances and *n*_*ij*_ represents the number of individual trees of the *j*^*th*^ provenance within the *i*^*th*^ block.

The genetic variation coefficient (*CV*_*G*_) was calculated using the following formula^[53]^:

7 $ CVG({\%} ) = 100 \times \frac{{\sqrt {{V_P}} }}{{\overline X }} $

Where $ \overline X$ is the average phenotypic mean of the trait, and *V*_*P*_ is the provenance variance.

The realized gain (G) was estimated by:

8 $ G = \frac{{({{\overline X}_i} - \overline X)}}{{\overline X}} \times 100{\%} $

Where $ {\overline X_i} $ and $ \overline X $ are the mean values of the selected superior provenance and the overall trait, respectively.

The genetic and phenotypic correlations between traits were calculated as follows:

9 $ {r_g} = \frac{{Co{v_{a1,a2}}}}{{\sqrt {{V_{a1}} \times {V_{a2}}} }} $

10 $ {r_P} = \frac{{Co{v_{a1,a2}} + Co{v_{e1,e2}}}}{{\sqrt {({V_{a1}} + {V_{e1}}) \times ({V_{a2}} + {V_{e2}})} }} $

Where *r*_*g*_ and *r*_*P*_ are genetic and phenotypic correlations, respectively, and* Cov*_*a1,a2*_ is the provenance covariance between traits *a*_*1*_ and *a*_*2*_. *Cov*_*e1,e2*_is the error covariance between traits *a*_*1*_ and *a*_*2*_, *V*_*a1*_ and *V*_*a2*_ are the provenance variances for trait *a*_*1*_ and *a*_*2*_, and *V*_*e1*_ and *V*_*e2*_ are residual error variances for traits *a*_*1*_ and *a*_*2*_.

Surfer 13.0 (Golden Software, Golden, CO, USA) software was used for trend surface mapping. The regression equation for the trend surface analysis was as follows:

11 $ Zi=\beta_{0}+\beta_{1}x+\beta_{2}y+\beta_{3}x_{2}+\beta_{4}y_{2}+\beta_{5}xy+\varepsilon_{ij}                                                    $

Where *β* is the regression coefficient, *x* is the latitude, *y* is the longitude, and *ε*_*ij*_ is the random error.
